# Is increasing complexity of algorithms the price for higher accuracy? virtual comparison of three algorithms for tertiary level management of chronic cough in people living with HIV in a low-income country

**DOI:** 10.1186/1472-6947-12-2

**Published:** 2012-01-19

**Authors:** Constance Mukabatsinda, Jasmine Nguyen, Bettina Bisig, Lutgarde Lynen, Yerma D Coppens, Anita Asiimwe, Jef Van den Ende

**Affiliations:** 1 Department of Internal Medicine, Centre Hospitalier Universitaire, Kigali, Rwanda; 2 Department of Clinical Sciences, Institute of Tropical Medicine, Antwerp, Belgium

**Keywords:** HIV, chronic cough, algorithms, clinical decision making, harm, complexity

## Abstract

**Background:**

The algorithmic approach to guidelines has been introduced and promoted on a large scale since the 1970s. This study aims at comparing the performance of three algorithms for the management of chronic cough in patients with HIV infection, and at reassessing the current position of algorithmic guidelines in clinical decision making through an analysis of accuracy, harm and complexity.

**Methods:**

Data were collected at the University Hospital of Kigali (CHUK) in a total of 201 HIV-positive hospitalised patients with chronic cough. We simulated management of each patient following the three algorithms. The first was locally tailored by clinicians from CHUK, the second and third were drawn from publications by Médecins sans Frontières (MSF) and the World Health Organisation (WHO). Semantic analysis techniques known as Clinical Algorithm Nosology were used to compare them in terms of complexity and similarity. For each of them, we assessed the sensitivity, delay to diagnosis and hypothetical harm of false positives and false negatives.

**Results:**

The principal diagnoses were tuberculosis (21%) and pneumocystosis (19%). Sensitivity, representing the proportion of correct diagnoses made by each algorithm, was 95.7%, 88% and 70% for CHUK, MSF and WHO, respectively. Mean time to appropriate management was 1.86 days for CHUK and 3.46 for the MSF algorithm. The CHUK algorithm was the most complex, followed by MSF and WHO. Total harm was by far the highest for the WHO algorithm, followed by MSF and CHUK.

**Conclusions:**

This study confirms our hypothesis that sensitivity and patient safety (i.e. less expected harm) are proportional to the complexity of algorithms, though increased complexity may make them difficult to use in practice.

## Background

The algorithmic approach to guidelines has been introduced and promoted on a large scale since the 1970s. This flowchart representation of step-by-step clinical logic guides the management of a patient with symptoms, clinical signs, or results of technical examinations. The transition from one step to the next is mostly dichotomous, which means that only one out of two choices can be made at each step. Moreover, the logic is serial: only one pathway can be followed by a single patient.

The original purpose of algorithmic guideline implementation was twofold. First, with continuing concern over the rising costs of health care, health policy makers have been impressed by the high levels of variation and inappropriateness in medical care. Second, in resource-poor settings, nurses provide frequently the first-line medical care. To deal with the relative lack of knowledge in this medical professional group, algorithms have been used to help rationalise and standardise clinical decision making, an approach considered so far to be effective and efficient [1]. Algorithms have been pivotal in the task-shifting or task-sharing by nurses that has been promoted over the last 10 years to increase access to antiretroviral therapy.

Clinical algorithms are often controversial: on the one hand, they have often been considered with suspicion because of their restrictive, "cookbook"-medicine approach. On the other hand, they have been viewed as a valuable educational tool, providing neophytes with a method for making diagnostic and therapeutic decisions in clinical practice apprenticeship [2]. Although both of these views may make sense, none of them has been validated.

Algorithms usually lead to a single diagnosis, potentially neglecting another serious one, leading to delayed investigations and treatment. In some situations, the burden of harm might grow considerably: when we follow an algorithmic guideline step-by-step, it is obvious to stop at the first diagnosis corresponding with the patient's findings. It induces a tunnel-vision and limits the search of certainty in diagnosis.

Epidemiology shows the relevance of the dynamics of the disease spectrum in time as well as in space. The utility of a given clinical algorithm can vary substantially across different epidemiologic and socioeconomic contexts. As a result, every algorithmic guideline must be adapted to every local context and regularly revised [3]. Moreover, the emphasis on development and deployment of medical guidelines has led to the introduction of multiple versions addressing the same problem: uncertainty is increased with the fact that clinicians have to select among several guidelines. Barak et al. tried to solve this problem by creating a method of text-to-algorithm conversion [2].

Several studies have evaluated or compared guidelines but none of them has compared them in terms of complexity or harm [4-7]. Wabitsch, in his efforts to achieve reproducible, clinically flexible and applicable guidelines, developed a systematic procedure to tailor guidelines to specific settings. Doing this, he compared two modified guidelines with a global one, but he did not consider harm [3]. Because of the impact guidelines may have on clinical practice, Pearson et al. proposed a method to develop, use and compare them [8]. This methodology-the Clinical Algorithm Nosology (CAN)-measures overall design complexity independent of algorithm content, quantitatively delineates algorithm differences, and qualitatively estimates the potential impact of such differences on patient care, by scoring the degree of similarity. This simple method may help clinicians to make the best choice between several algorithms about the same subject or complaint in their setting.

At the end of 2007 the global estimate of the number of people living with HIV was about 33,2 million, with more than 96% in low- and middle-income countries [9]. In Rwanda, HIV prevalence turns around 2,9 to 3,2% [10].Except for some countries, the majority of people living with HIV still have no access to highly active antiretroviral therapy (HAART). Chronic cough is a key problem of medical decision making in the management of immune-compromised HIV patients, since a large cluster of dangerous but treatable diseases present this way.

The purpose of this research was to compare virtually three algorithmic guidelines intended for use at a tertiary level in terms of sensitivity, delay to diagnosis, harm, complexity and similarity. We intended to evaluate how complexity relates to accuracy and expected harm. We hypothesized that increasing complexity, thereby approaching the clinician's intuitive logic, improves accuracy and diminishes expected harm, but that, on the other hand, the degree of complexity can become too high and make the algorithm less useful in practice.

## Methods

### Patients

Data were collected at the University Hospital of Kigali (CHUK), a national reference centre for Rwanda, during a 4 months period in 2002. The study included all adults (> 15 years of age) hospitalised in the internal medicine department with cough of more than three weeks' duration as their chief complaint, known or newly confirmed HIV-positive and suspected of World Health Organisation (WHO) stage 3 or 4 [11]. Patients who had a known non-infectious cause of chronic cough (chronic obstructive lung disease, bronchial asthma, pleural effusion due to cardiac or renal insufficiency) were excluded from the study. The reference standard for the actual disease of each patient was the final diagnosis made by clinicians at the end of the follow-up with the available diagnostic tools (e.g., chest X-ray, Gram and Ziehl-Neelsen stain, culture; bronchoscopy was done for selected cases).

Clinical care depended only on the clinician's judgment. Moreover, clinicians were blinded to hypothetical results of the application of the algorithms.

For each patient, we collected data through a questionnaire in Epi-Info software 2000: age, sex, history, physical examination, tests done, treatment, outcome and final diagnosis. Data were made anonymous for analysis. Sensitivity was computed with and without considering patients for whom no final diagnosis was made and those with a rare diagnosis not included in the algorithms. For the remainder of the analysis they were excluded.

### Algorithms

**Figure 1:**
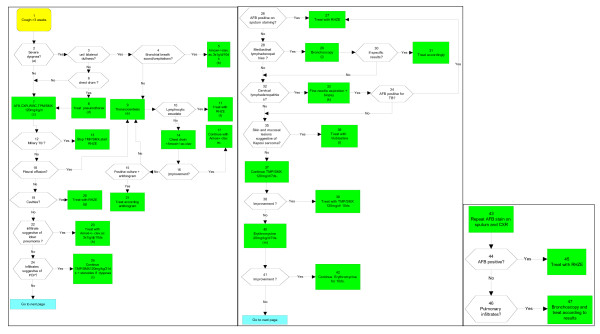
**THE CHUK ALGORITHMIC GUIDELINE**.

Using the graphic format described by Margolis, a serial and dichotomous flowchart representation adopted by the Harvard Community Health Plan, a locally tailored algorithm was developed based on clinicians' logic and availability of complementary exams (Figure 1). Two residents, under the supervision of their professor of internal medicine, created the CHUK algorithm. They were not amongst the clinicians who made the final diagnosis considered as the gold standard. Clinicians only controlled the construction of the algorithm for inconsistencies.

**Figure 2:**
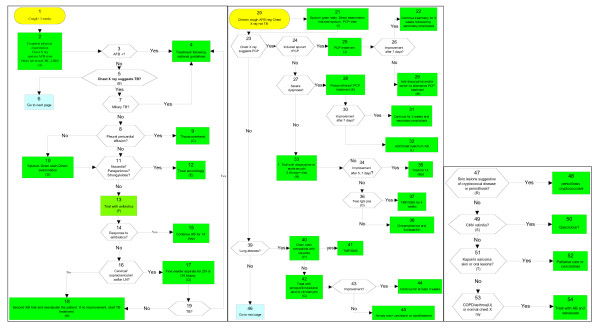
**THE MSF ALGORITHMIC GUIDELINE**.

**Figure 3:**
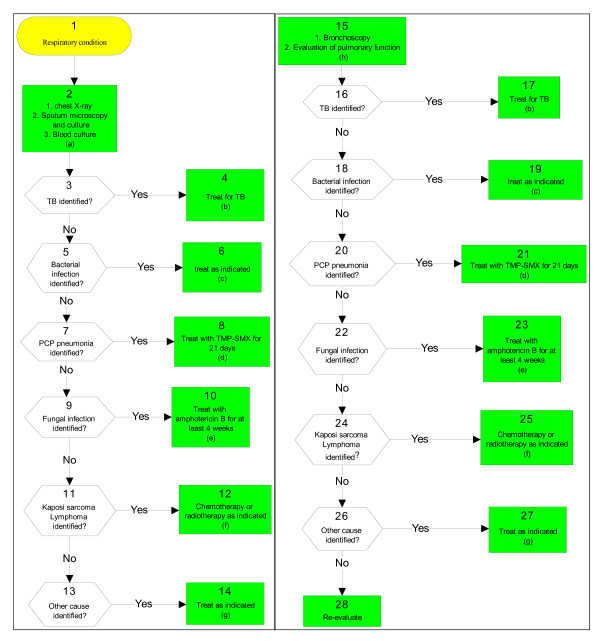
**THE WHO ALGORITHMIC GUIDELINE**.

The Médecins sans Frontières (MSF) algorithm was published in 2001 by L. Lynen for management of opportunistic infections in HIV-positive patients, and based on an extensive literature review (Figure 2) [12,13]. The WHO algorithm was published earlier in the 1990s (Figure 3) [14]. Both publications proposed guidelines for care at three levels. In our assessment of the three algorithms we only used the chronic cough algorithm for the tertiary care level.

### Evaluation

We virtually applied the three algorithms to each patient, using data from the database, simulating a step-by-step management as would have been done following the algorithm. We calculated the number of patients who found a way through the algorithm ("fit") and we compared their management in the ward with the pathway they would have followed with the algorithm. Sensitivity represents the proportion of correct diagnosis made by an algorithm. We estimated the sensitivity first for each diagnosis, and further for the algorithm as a whole, with the final clinical diagnosis as the "true condition". If the algorithm missed a diagnosis, we considered this as a false negative for the true condition. If another diagnosis was reached instead of the true condition, it was considered as a false negative for the true condition and as a false positive for the wrong condition. In other words, the false positive is a wrong diagnosis whereas the false negative is a missed diagnosis.

Starting from the clinical state box to the action box, we defined for each diagnosis a minimal hypothetical (ideal) delay, depending on the execution of the different investigations. For every patient we compared the time clinicians needed in the ward to reach a therapy, with the delay predicted by the different algorithms. Delays were considered on theoretical grounds, supposing that all steps had to be taken in a sequential way (which clinicians often don't do: e.g., if the X-ray shows a miliary pattern, they will not wait for all three sputa examinations).

We compared the algorithms following the Clinical Algorithm Nosology. It is made up of two components [8]. The Clinical Algorithm Structural Analysis (CASA) measures overall design and flow pattern complexity independent of algorithm content. A formula expresses the relative complexity of a clinical algorithm in a score:

$$“\text{CASA}\;\text{complexity}\;\text{score}”\;=\;2\;\left({\text{n}}_{1}{\text{D}}_{\text{x}}\right)\;+\;1\;\left({\text{n}}_{2}{\text{D}}_{0}\right)\;+\sum \;{\left({\text{L}}_{\text{p}}\right)}_{\text{i}}$$

where n_1_D_x _= number of diagnostic boxes, n_2_D_0 _= number of all other boxes, and ∑ (L_p_) _i _= the summation of "loop parameters" (i.e. nodes that send the clinician back to an earlier point). The Clinical Algorithm Patient Abstraction (CAPA) then accomplishes a clinical comparison of two algorithms and explains the differences between them. The CAPA has three steps: clinical rule analysis, patient abstraction and patient comparison. "Identical" (score = 10) means same order of clinical exams and decision, "similar" (score = 8) means the same steps and treatment but in different order and "different" (score = 0) means a management without the same diagnosis or therapeutic decision.

With a questionnaire featuring scoring scales from 0 to 10, nine internal medicine clinicians of CHUK estimated intuitively the weight of harm by commission (false positives) and harm by omission (false negatives) in therapeutic decisions compared to natural death [15]. We relied only on intuitive estimations, after a training session explaining the different key factors determining harm: disease and treatment morbidity and mortality, treatment efficacy and cost, stigma, contagiousness. For each algorithm we computed the harm per diagnosis, multiplying false positives and false negatives with their respective harm, and summing these for the overall harm.

## Results

Data were recorded for 201 patients. Nine patients had no diagnosis, 9 had a rare diagnosis not included in the algorithms, leaving 183 for analysis. One patient had two probable diagnoses. Sex ratio was 82/119 M/F (0,69), mean age was 35,9 years (range 15-70 years). Baseline characteristics and main diagnostic frequencies are summarized in table 1. (Table 1) All forms of tuberculosis were by far the most frequent diagnosis (46,4%). Mortality was high (39%).

**Table 1:** baseline characteristics of patients (n = 201).

Age (years)	Count	%
15-24	22	11
25-34	72	36
35-44	69	34,5
45-54	29	14
55-64	8	4
65-74	1	0,5

**sex**		

male	82	41
female	119	59

**Diagnosis**		

TB smear positive	43	21,4
Pneumocystis jirovecii	39	19,4
Pleural TB	25	12,4
Lobar pneumonia	21	10,4
Miliar TB	18	9
Atypical pneumonia	18	9
Kaposi 's sarcoma	7	3,5
Empyema	3	1,5
Pulmonar abscess	3	1,5
Mediastinal adenopathy (ADP) TB	3	1,5
Cavitary TB	2	1
Pericardial TB	2	1
Other diagnosis*	9	4,5
no diagnosis	9	4,5

Other diagnosis: encephalitis, other forms of extra pulmonary TB x 4, (newly diagnosed) heart failure, haemolytic anaemia, pneumothorax, cryptococcal meningitis.

Of the 184 cases (one patient with 2 diagnoses) we made follow the CHUK algorithm, 183 fit a pathway (99,5%). Comparing the algorithm pathways with the one followed by the clinicians, for 61% the management was "identical", for 38% it was "different" and for 1% it was "similar".

The same procedure was also applied for each of the 184 cases to the other two algorithms under study. A fit was found in 183 (99.5%), 183 (99.5%), and 171 (92.9%) cases for the CHUK, MSF and WHO algorithms, respectively. Sensitivity was 95.7%, 88% and 70.1% for the CHUK, MSF and WHO algorithms, respectively (87.5%, 80.5% and 64.2 while including patients with unknown and rare diagnosis). (Table 2)

**Table 2:** details of accuracy of three algorithms.

Diagnosis	N°	algo CHK					algo MSF					algo OMS				
		**false pos**	**true pos**	**false neg**	**true neg**	**fit**	**false pos**	**true pos**	**false neg**	**true neg**	**fit**	**false pos**	**true pos**	**false neg**	**true neg**	**fit**
TB BK+	43	1	42	1	140	43	0	43	0	141	43	0	43	0	141	43
miliary TB	18	0	18	0	166	18	0	18	0	166	18	0	18	0	166	18
pleural TB	25	0	24	1	159	25	0	25	0	159	25	0	3	22	159	24
PCP	39	0	39	0	145	39	0	39	0	145	39	0	39	0	145	39
lobar pneumonia	21	3	20	1	160	21	0	1	20	163	21	0	1	20	163	21
atypical pneumonia	18	1	17	1	165	18	20	18	0	146	18	20	18	0	146	18
empyema	3	0	3	0	181	3	0	3	0	181	3	0	0	3	181	0
Kaposi	7	0	7	0	177	7	0	7	0	177	7	0	7	0	177	7
pulmonal abcess	3	0	0	3	181	2	1	3	0	180	3	0	0	3	181	0
cavitary TB	2	1	2	0	181	2	0	0	2	182	1	0	0	2	182	0
TB ADP mediastinal	3	1	2	1	180	3	0	3	0	181	3	0	0	3	181	0
TB pericarditis	2	0	2	0	182	2	0	2	0	182	2	0	0	2	182	1

**globally**	**184**	7	176	8	2017	183	21	162	22	2003	183	20	129	55	2004	171

Table 3 shows that for most diagnoses a certain delay to appropriate management was present. (Table 3) For example, for empyema, seven days were lost by the CHUK clinicians before a therapeutic action. When we compare the average delay of CHUK and MSF guidelines with the hypothetical minimal delay according to the local situation regarding availability and speed of execution of tests and treatments, the CHUK algorithm was the fastest, followed by that of MSF. For the WHO algorithm, delays were difficult to calculate because of missing practical specifications.

**Table 3:** theoretical delay in days if tests were performed in a serial way.

Final Clinical Diagnosis	CHUK	MSF	Actual delay in CHUK	Hypothetical minimal delay
TB smear positive	3,14	2,91	2,3	3
Miliary TB	1	3	1,6	1
Pleural TB	2,28	4	3,4	2
Pneumocystis jirovecii	1,03	3,45	0	1
Lobar pneumonia	1,09	3,09	0,8	1
Atypical pneumonia	1,67	4,83	0,2	1
Empyema	1,67	3,67	6,7	2
Kaposi 's sarcoma	1,57	3,14	1,6	1
Pulmonary abscess	1	3	1	1
Cavitary TB	1,5	3	0	1
Mediastinal ADP TB	4,33	6	3	1
Pericardial TB	2	4	0	1

**Average delay**	**1,86**	**3,46**	**1,8**	**1,3**

The CHUK algorithm was the most complex with a CASA score of 80, followed by MSF with a score of 73 and WHO with a score of 40. The CAPA score was 0 meaning that there is (almost) no similarity between the three algorithms.

Table 4 shows the average intuitive harm for false positives and negatives for each disease, weighted by the nine internal medicine clinicians compared to natural death. On average, the weight of omission error was much higher than the weight of commission error (8 versus 4.5). The total harm was the highest for the WHO algorithm with a score of 487.5. On the other hand, the CHUK algorithm was by far the least harmful followed by MSF algorithm with scores of 91 and 240, respectively. If we split total harm into "false-positive harm" and "false-negative harm", we obtain, respectively, for CHUK scores of 27 and 63, for MSF 64 and 176 and for WHO 60 and 427.

**Table 4:** estimation of intuitive harm.

Diagnosis	False Negatives	False Positives
TB smear positive	9	5
Miliar TB	8,5	5
Pleural TB	7,5	4,5
Pneumocystis jirovecii	8	3
Lobar pneumonia	8	3
Atypical pneumonia	7	3
Empyema	8	4
Kaposi 's sarcoma	7	7,5
Pulmonar abscess	8	4
Cavitary TB	8	5
Mediastinal ADP TB	7,5	5
Pericardial TB	8	5

Average	8	4,5

Average of estimation of intuitive harm of false negatives and false positives for each diagnosis. Nine internists scored on a scale from 0 to 10.

## Discussion

### Results

The purpose of this study was to assess the hypothetical utility of three algorithms in terms of sensitivity, delay to action and harm at a tertiary care level. The CHUK guidelines proved to be the most complex, but the most sensitive and the least harmful. The results confirm that increasing complexity improves sensitivity and diminishes expected harm. But the degree of complexity becomes so high that it might be one of the reasons why clinicians are reluctant to use them. As a matter of fact, none of these algorithms has ever been used in daily practice in CHUK.

### Epidemiology

Due to limited diagnostic means, the gold standard in this study is the clinical diagnosis made by the clinicians at CHUK. This might be considered as a weakness. However, the prevalence of final diagnoses is close to the spectrum of diseases that cause chronic cough in people living with HIV in Africa [10]: tuberculosis remains the main diagnosis, found in 46% of cases in our study. It is now well described that tuberculosis is the leading opportunistic infection in most low-resource settings, unlike industrialised countries in which a preponderance of *Pneumocystis jirovecii *(formerly *carinii*) is seen. Our study reveals a prevalence of 19% of pneumocystosis. This is fourfold the prevalence found by Batungwanayo in the same hospital ten years earlier, but this result was based on microbiological evidence after bronchoscopy and was restricted to unsolved cases [16]. In Uganda bronchoscopy in smear negative patients showed pneumocystosis in 38% [17]. Since in our study the diagnosis of pneumocystosis was made mainly radiologically, and since we consider all patients with persistent cough, these data are difficult to compare. A review in Thailand of causes of all respiratory symptoms in HIV infected patients found 23% cases of pneumocystosis, a rate more similar to our data [18].

In our study, mortality rate was high (39%). At that moment in time, ART was not subsidized; patients feared hospitalisation in our wards because they knew beforehand the high mortality, leading to late referral or consultation. Beyond this, some deaths could be attributed to missed diagnosis of uncommon diseases.

### Delay

The average delay to appropriate management would be shorter for the CHUK algorithm (1.8 days) than for the MSF (3.4 days). The CHUK delay is therefore closer to that seen in clinical practice at CHUK, as well as to the ideal one (Table 3). The theoretical differences between CHUK and MSF algorithms can be explained by the sequence of investigations: CHUK algorithm focused first on an X-ray to diagnose urgent pathologies, while MSF first focuses on sputum smears to diagnose the most frequent pathology (TB). In practice the X-ray results are read on day 1 while the sputa are being analyzed.

The delay of the WHO algorithm was difficult to evaluate because this guideline is mostly theoretical and gives no practical details: reading it seems simple, but to put it into practice is more complex and becomes impossible for several diagnoses, leaving a lot of freedom to the clinician. To illustrate this, there is no way to distinguish lobar and atypical pneumonia; in pleurisy, they recommend doing a tap, but there is no explanation about the interpretation of the results or about steps to take.

### Sensitivity, harm and complexity

The analysis of the algorithms brings to the fore that the CHUK algorithm is the one that leads to the highest sensitivity and induces the lowest harm in terms of morbidity and mortality. This is probably because we tried to come as close as possible to the intuitive reasoning of the experienced CHUK clinicians. The algorithm was developed by trainees of the CHUK clinicians, and CHUK clinicians are used in this analysis as the gold standard. On the other hand, it is by far the most complex with a high CASA score of 80. Part of the high complexity in the CHUK algorithm is explained by the presence of many loops. We see a clear inverse relation between the increasing sensitivity and the decreasing harm for all three algorithms. In addition, with regards to the sensitivity we note that despite such a complexity, 8 patients (4,3%) would not reach a correct final diagnosis.

### Weaknesses

#### Epidemiology

Firstly we noticed that in the set of patients and also in the different diagnoses available in the CHUK algorithm, uncommon but treatable diseases such as aspergillosis, nocardiosis, cryptococcosis, and some others are not taken into account. These diseases, however, are well described as opportunistic infections in HIV-positive patients and deserve priority before considering frequent diseases because of their seriousness and treatability. We omitted them from the analysis since algorithms cannot cover all rare diseases.

Secondly, in current practice in low resource settings, diagnosis of pulmonary infection is often a presumption, based on clinical signs and radiological results because of the limited availability of serology, culture or PCR. These presumptive diagnoses were considered as gold standard-not always with formal proof. It casts doubt on the reliability of the comparison of the diagnosis obtained by following the algorithm with the gold standard diagnosis, as the latter may have been false. The ideal situation should offer availability of complementary exams to confirm these presumptive diagnoses, but it would imply submitting patients to invasive procedures and spending much more money. Moreover, in advanced AIDS, more than one pathogen is frequently identified [18,19].

Finally, a large part of the difference in sensitivity of the CHUK and MSF algorithms is due to one single step differentiating between lobar and atypical pneumonia.

#### Algorithms and CAN

The three algorithms might not be comparable: the WHO algorithm leaves much to the discretion of the clinician, without specifications.

The CAN methodology can be applied easily. By itself, however, it does not judge which of the algorithms is likely to provide better patient care. This sole method of comparison is not really used in practice and remains an elusive concept. It is only a comparative tool to quantify the degree of similarity and complexity with no attention for sensitivity and harm which is the most important quality of a diagnostic aid. The authors mention that the ideal objective in the comparison of algorithms would involve decision analyses of the management pathways and prospective clinical trials comparing patient outcome in guided care [8]. Decision analysis would include sensitivity analysis of sensitivity and specificity of every finding for every concerned disease, of different sequences of nodes, and expressing global harm in DALY's or QUALI's for every disease. An almost impossible task, at least for complex algorithms as the ones discussed here. On the other hand, prospective clinical trials would involve huge sample sizes, as for every disease a sufficient number of patients should be included. Moreover, ethical requirements should be met.

So far, a practical way of comparing clinical guidelines proving their reliability before their implementation is still needed to validate any guideline program [8]. In our study, we did not apply algorithms in real patients and evaluate the outcome. We consider that our methodology is anyhow a necessary first step in validation: a correct scientific output is reached without the risk of increasing harm applying algorithms in real clinical decision making.

#### Estimation of harm

Intuitive weighting of harm is a gross intuitive estimation given by clinicians on a simple scale scoring from 0 to 10 about commission error (false positives) and omission error (false negatives) in therapeutic decisions. Calculated weighed harm is more accurate than intuitive weighed harm, because it is based on observed probabilities related with the outcome of treated and non treated diseased and non diseased patients. In a recent study, we proved that the ratio of the intuitively estimated harm was almost 32 times lower than the calculated based on (by the same clinicians) intuitively estimated influencing factors, and two times lower than the calculated weighed harm based on literature data [20]. For our study and purpose, we relied only on the intuitive one to draw conclusions, conscious of the lack of accuracy.

### Impact

Our study has shown that some published algorithms have limited accuracy. This can be overcome by increasing the complexity, at the possible expense of user-friendliness. We hope that this study encourages policymakers to "dry-run" new draft-algorithms with an existing database of a cohort of patients, before organizing a formal validation in a real situation. The latter is not always possible, given the lack of a gold standard in most situations where the algorithm is to be applied.

A second impact of this study might be that writing algorithms for problems with a large differential diagnosis at the tertiary care level should be abandoned for many reasons, for instance: the burden of work necessary to adapt the algorithm to time and place, the neglect of rare but serious and treatable diseases, the possible coexistence of several pathologies, the reduction of a clinical presentation to only a few signs, and the difficulty in reaching a reasonable accuracy. We might state that at this level of care, medical decision tools based on a serial dichotomous approach may be replaced by the more human parallel and weighed logical approach recently proposed [20-23].

### Future research

Little research has been done to evaluate the real-world use of algorithms by health professionals [1,3]. Anecdotal evidence shows that most nurse practitioners do not follow their algorithms. As far as we are concerned, doctors never follow them for diagnosis; simple therapeutic flow-charts on the other hand, do well.

The concept of threshold approach in clinical decision-making has existed since the 1970s, and this concept has been substantiated to the point that ignoring it would be unethical [24-26]. All the more, a threshold approach might solve many conflicts provoked by the algorithmic logic: future studies will have to demonstrate it.

Considering that application of an algorithmic approach involves a short course training, especially for nurses and inexperienced personnel, future studies should assess which logical approach is the most effective for a given amount of training [23].

## Conclusion

Inexperienced medical staff could go on using the algorithmic approach in order to solve simple problems at the first level of care. The condition is that they should have a short basic training and that the flowchart should be validated, adapted to the local context and regularly revised. But at the tertiary care level, approaching the competence of specialised clinicians by improving sensitivity and diminishing expected harm will increase complexity at that point that it turns out almost impossible to apply.

## Competing interests

The authors declare that they have no competing interests.

## Authors' contributions

CM contributed to the concept, data collection and analysis, JN contributed to analysis and writing, BB contributed to concept and analysis, LL contributed to analysis and supervision of writing, YC contributed to data collection and analysis, AA contributed to concept, data collection and analysis, JVDE contributed to concept, analysis, writing and overall supervision. All authors read and approved the final manuscript.

## Pre-publication history

The pre-publication history for this paper can be accessed here:

http://www.biomedcentral.com/1472-6947/12/2/prepub

## References

[B1] Haegeman F Ledecq JL Wyffels A Dama K [Evaluation of the use of diagnostic/treatment algorithms in the health centers of North Cameroon] Ann Soc Belg Med Trop 1994 74 231 247 7840690

[B2] Barak N Margolis CZ Gottlieb LK Text-to-algorithm conversion to facilitate comparison of competing clinical guidelines Med Decis Making 1998 18 304 310 10.1177/0272989X9801800308 9679995

[B3] Wabitsch R Margolis CZ Malkin JE Abu-Saad K Waksman J Evaluating the process of tailoring the global HIV/AIDS practice guidelines for use in developing countries Int J Qual Health Care 1998 10 147 154 10.1093/intqhc/10.2.147 9690888

[B4] Saranchuk P Boulle A Hilderbrand K Coetzee D Bedelu M van CG Meintjes G Evaluation of a diagnostic algorithm for smear-negative pulmonary tuberculosis in HIV-infected adults S Afr Med J 2007 97 517 523 17805454

[B5] Ferrand RA Weiss HA Nathoo K Ndhlovu CE Mungofa S Munyati S Bandason T Gibb DM Corbett EL A primary care level algorithm for identifying HIV-infected adolescents in populations at high risk through mother-to-child transmission Trop Med Int Health 2011 16 349 355 10.1111/j.1365-3156.2010.02708.x 21176006 PMC3132444

[B6] Horwood C Vermaak K Rollins N Haskins L Nkosi P Qazi S Paediatric HIV management at primary care level: an evaluation of the integrated management of childhood illness (IMCI) guidelines for HIV BMC Pediatr 2009 9 59 10.1186/1471-2431-9-59 19772599 PMC2754450

[B7] Horwood C Liebeschuetz S Blaauw D Cassol S Qazi S Diagnosis of paediatric HIV infection in a primary health care setting with a clinical algorithm Bull World Health Organ 2003 81 858 866 14997238 PMC2572386

[B8] Pearson SD Margolis CZ Davis S Schreier LK Gottlieb LK The clinical algorithm nosology: a method for comparing algorithmic guidelines Med Decis Making 1992 12 123 131 10.1177/0272989X9201200205 1573979

[B9] UNAIDS HIV and AIDS estimates of 2007 2008 UNAIDS

[B10] ONUSIDA/OMS Le point sur l'épidémie de SIDA 2005 ONUSIDA/OMS

[B11] WHO Interim proposal for a WHO Staging System for HIV infection and Disease Wkly Epidemiol Rec 1990 65 221 224 1974812

[B12] Lynen L Clinical AIDS Care Guidelines For Resource poor Settings 2001 MSF Belgium-Luxembourg

[B13] Lynen L Treatment and Prevention of opportunistic infections at the level of Referral Hospital in Resource-limited Settings 2005 MSF Belgium-Luxembourg

[B14] WHO WHO, Guidelines for the clinical management of HIV infection in adult 2008 WHO/GPA/IDS/HCS/91.6

[B15] Tsalatsanis A Hozo I Vickers A Djulbegovic B A regret theory approach to decision curve analysis: a novel method for eliciting decision makers' preferences and decision-making BMC Med Inform Decis Mak 2010 10 51 10.1186/1472-6947-10-51 20846413 PMC2954854

[B16] Batungwanayo J Taelman H Lucas S Bogaerts J Alard D Kagame A Blanche P Clerinx J van de PP Allen S Pulmonary disease associated with the human immunodeficiency virus in Kigali, Rwanda. A fiberoptic bronchoscopic study of 111 cases of undetermined etiology Am J Respir Crit Care Med 1994 149 1591 1596 8004318 10.1164/ajrccm.149.6.8004318

[B17] Worodria W Okot-Nwang M Yoo SD Aisu T Causes of lower respiratory infection in HIV-infected Ugandan adults who are sputum AFB smear-negative Int J Tuberc Lung Dis 2003 7 117 123 12588011

[B18] Mootsikapun P Chetchotisakd P Intarapoka B Pulmonary infections in HIV infected patients J Med Assoc Thai 1996 79 477 485 8855629

[B19] Martinson NA Karstaedt A Venter WD Omar T King P Mbengo T Marais E McIntyre J Chaisson RE Hale M Causes of death in hospitalized adults with a premortem diagnosis of tuberculosis: an autopsy study AIDS 2007 21 2043 2050 10.1097/QAD.0b013e3282eea47f 17885294

[B20] Moreira J Bisig B Muwawenimana P Basinga P Bisoffi Z Haegeman F Kishore P Van den Ende J Weighing harm in therapeutic decisions of smear-negative pulmonary tuberculosis Med Decis Making 2009 29 380 390 10.1177/0272989X08327330 19224870

[B21] Van den Ende J Bisoffi Z Van Puymbroek H Van der SP Van Gompel A Derese A Lynen L Moreira J Janssen PA Bridging the gap between clinical practice and diagnostic clinical epidemiology: pilot experiences with a didactic model based on a logarithmic scale J Eval Clin Pract 2007 13 374 380 10.1111/j.1365-2753.2006.00710.x 17518802

[B22] Van Puymbroeck H Remmen R Denekens J Scherpbier A Bisoffi Z Van den Ende J Teaching problem solving and decision making in undergraduate medical education: an instructional strategy Med Teach 2003 25 547 550 14522680 10.1080/0142159031000137490

[B23] Bisoffi Z Sirima BS Angheben A Lodesani C Gobbi F Tinto H Van den Ende J Rapid malaria diagnostic tests vs. clinical management of malaria in rural Burkina Faso: safety and effect on clinical decisions. A randomized trial Trop Med Int Health 2009 14 491 498 10.1111/j.1365-3156.2009.02246.x 19222821

[B24] Pauker SG Kassirer JP The threshold approach to clinical decision making N Engl J Med 1980 302 1109 1117 10.1056/NEJM198005153022003 7366635

[B25] Pauker SG Pauker SP Wong JB The utility of thresholds and the threshold utility Med Decis Making 1987 7 201 202 10.1177/0272989X8700700401 3683106

[B26] Kassirer JP Our stubborn quest for diagnostic certainty. A cause of excessive testing [see comments] N Engl J Med 1989 320 1489 1491 10.1056/NEJM198906013202211 2497349

